# Spatiotemporal scaling changes in gait in a progressive model of Parkinson's disease

**DOI:** 10.3389/fneur.2022.1041934

**Published:** 2022-12-13

**Authors:** Alex M. Doyle, Devyn Bauer, Claudia Hendrix, Ying Yu, Shane D. Nebeck, Sinta Fergus, Jordan Krieg, Lucius K. Wilmerding, Madeline Blumenfeld, Emily Lecy, Chelsea Spencer, Ziling Luo, Disa Sullivan, Krista Brackman, Dylan Ross, Sendréa Best, Ajay Verma, Tyler Havel, Jing Wang, Luke Johnson, Jerrold L. Vitek, Matthew D. Johnson

**Affiliations:** ^1^Department of Neuroscience, University of Minnesota, Minneapolis, MN, United States; ^2^Department of Neurology, University of Minnesota, Minneapolis, MN, United States; ^3^Department of Biomedical Engineering, University of Minnesota, Minneapolis, MN, United States

**Keywords:** Parkinson's disease, gait, MPTP, non-human primate, pressure walkway

## Abstract

**Objective:**

Gait dysfunction is one of the most difficult motor signs to treat in patients with Parkinson's disease (PD). Understanding its pathophysiology and developing more effective therapies for parkinsonian gait dysfunction will require preclinical studies that can quantitatively and objectively assess the spatial and temporal features of gait.

**Design:**

We developed a novel system for measuring volitional, naturalistic gait patterns in non-human primates, and then applied the approach to characterize the progression of parkinsonian gait dysfunction across a sequence of 1-methyl-4-phenyl-1,2,3,6-tetrahydropyridine (MPTP) treatments that allowed for intrasubject comparisons across mild, moderate, and severe stages.

**Results:**

Parkinsonian gait dysfunction was characterized across treatment levels by a slower stride speed, increased time in both the stance and swing phase of the stride cycle, and decreased cadence that progressively worsened with overall parkinsonian severity. In contrast, decreased stride length occurred most notably in the moderate to severe parkinsonian state.

**Conclusion:**

The results suggest that mild parkinsonism in the primate model of PD starts with temporal gait deficits, whereas spatial gait deficits manifest after reaching a more severe parkinsonian state overall. This study provides important context for preclinical studies in non-human primates studying the neurophysiology of and treatments for parkinsonian gait.

## Introduction

Parkinson's disease is characterized by several cardinal motor signs that include bradykinesia, resting tremor, muscle rigidity, and gait and postural control dysfunction (1, 2). Of these motor signs, gait dysfunction is often the most debilitating in terms of quality of life (3–6) and can be the most difficult to treat with dopaminergic drugs or with deep brain stimulation (DBS) therapy (7–10). Parkinsonian gait dysfunction in humans consists of reduced gait speed, decreased stride length (distance between two consecutive heel steps of the same foot), increased cadence, and variability in gait patterns (10–12).

Given the clinical need for more robust treatments of parkinsonian gait dysfunction, further study is needed to better understand the pathophysiology of parkinsonian gait (13) and the neurophysiological changes (14) that occur within the brain with treatments that improve one or more aspects of parkinsonian gait. Animal models of PD provide an opportunity to not only explore brain circuits underlying parkinsonian gait deficits but to also investigate and further develop new therapies for treating these deficits (15, 16). Rodent studies have demonstrated that 6-OHDA and MPTP treatments induce shorter stride lengths (17–20), increased time in the stance phase, and decreased time in the swing phase (17, 19, 20). Studies looking at dynamic components of gait have also found increased variability in gait measures (17, 19) and asymmetry of gait (21).

The systemic MPTP model of PD in aged non-human primates (NHPs) has been shown to mimic many of the gait deficits observed in human PD including decreased gait speed, stride length, and stride speed (22–24). To date, analysis of gait dysfunction in this animal model of PD have included subjective ratings through motor-sign criteria based on clinical rating scales (25–27) or video analysis of subjects walking while being guided using a pole-and-collar system (22, 23). Additional investigations include NHPs in a non-PD state that have been trained to walk bipedally on a treadmill while restrained within a recording apparatus to investigate neuronal activity in the brainstem during gait (28). While much has been discovered from these studies, natural ambulatory behavior for NHPs is quadrupedal walking (29, 30) without physical restraint. To that end, a recent study adapted a commonly used murine automated gait analysis stem to quantify commonly studied gait parameters in parkinsonian marmosets (31). However, there remains a need to investigate natural ambulatory behavior and how gait dysfunction evolves with increasing parkinsonian severity.

Clinical studies investigating parkinsonian gait often provide quantitative analysis of gait parameters using, for example, a pressure walkway system enabling unrestricted ambulation between spatial locations (32–36). Such an approach allows one to quantify gait initiation and turning, which are known to be affected in PD (37, 38). Rodent studies have utilized similar quantitative gait analysis methods including optical imaging of a glass walkway plate to capture the static and dynamic components of gait and pressure intensity of limb contacts (20, 39). Developing a similar quantitative approach for parkinsonian NHPs would enable future studies to dissect the cortical and subcortical pathophysiological changes that underlie parkinsonian gait dysfunction and to investigate targeted treatments including DBS therapy.

In this study, we developed a novel testing apparatus to quantitatively assess gait parameters of freely-ambulating NHPs. We describe quantitative spatiotemporal analysis of changes in gait parameters across multiple severity levels of parkinsonism in the MPTP-treated NHP model of PD. These quantitative changes were then compared and evaluated in the context of known gait dysfunction parameters in human PD.

## Methods

### Animals

Kinematic gait data were collected from three adult female rhesus macaques (*Macaca mulatta*; Subject A: 24 yrs. old, 10 kg; Subject B: 23 yrs. old 12 kg; Subject N: 16 yrs. old, 11 kg). Animal care complied with the National Institutes of Health Guide for the Care and Use of Laboratory Animals and all behavioral protocols were approved by the University of Minnesota Institutional Animal Care and Use Committee.

### MPTP administration

**Figure 1:**
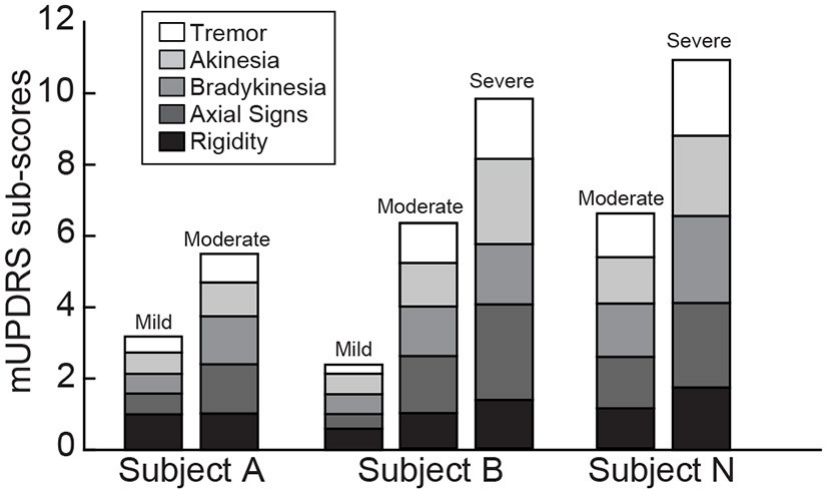
mUPDRS sub-score evaluations of cardinal motor signs following MPTP treatments in each subject. Evaluations were conducted in the mild parkinsonian state for two subjects (B, A), moderate parkinsonian state for all subjects, and severe parkinsonian state for two subjects (B, N). mUPDRS sub-scores included evaluation of the most affected side of the body for tremor, akinesia, bradykinesia and rigidity, plus whole-body axial motor signs. The span of each bar represents the sub scores and the height reflects the total additive scores.

To investigate gait parameter changes between naïve and parkinsonian states, all subjects were rendered parkinsonian with systemic intramuscular injections of MPTP (Sigma-Aldrich and Toronto Research Chemicals, ~0.2–0.4 mg/kg per dose) (40–43). MPTP cumulative dosage and injections for each parkinsonian state is documented in Table 1. The mild, moderate, and severe parkinsonian states were quantified by assessing the severity of parkinsonian motor signs using the modified Unified Parkinson's Disease Rating Scale (mUPDRS) (44–46) (Figure 1). The motor sign categories included bradykinesia, akinesia, rigidity, tremor, and axial motor control dysfunction and were evaluated on a scale from 0 to 3 in the 5 subscore categories with three being severely affected. Axial motor scores were calculated as an average rating of gait, posture, balance, and turning dysfunction. Total mUDPRS score ranges for the mild (1–5), moderate (5–10), and severe (10–15) PD states were scaled from previously published ranges containing more categories (40–43).

**Table 1:** Summary of MPTP dosing over time.

	**Mild parkinsonism**			**Moderate parkinsonism**			**Severe parkinsonism**		
**Subject**	**Cumulative dosage**	**# of injections**	**Period**	**Cumulative dosage**	**# of injections**	**Period**	**Cumulative dosage**	**# of injections**	**Period**
	**(mg/kg)**		**(days)**	**(mg/kg)**		**(days)**	**(mg/kg)**		**(days)**
A	1.7	6	2	2.3	2	2	.	.	.
B	1.1	4	36	3.9	12	98	12.14	31	252
N ^¥^	.	.	.	1.2	1	1	0.8	2	2

¥ An MPTP injection induced moderate parkinsonism in Subject N bypassing the mild parkinsonian state. Doses for subject A (seven injections 0.3 mg/kg, one injections 0.23 mg/kg), subject B (27 injections 0.2 mg/kg, 14 injections 0.3 mg/kg, and six injections 0.4 mg/kg), subject N (three injections 0.4 mg/kg). Cumulative MPTP dosage, number of injections, and time period needed to reach each parkinsonian state. Cells with “.” indicate unavailable data.

### Gait testing apparatus

**Figure 2:**
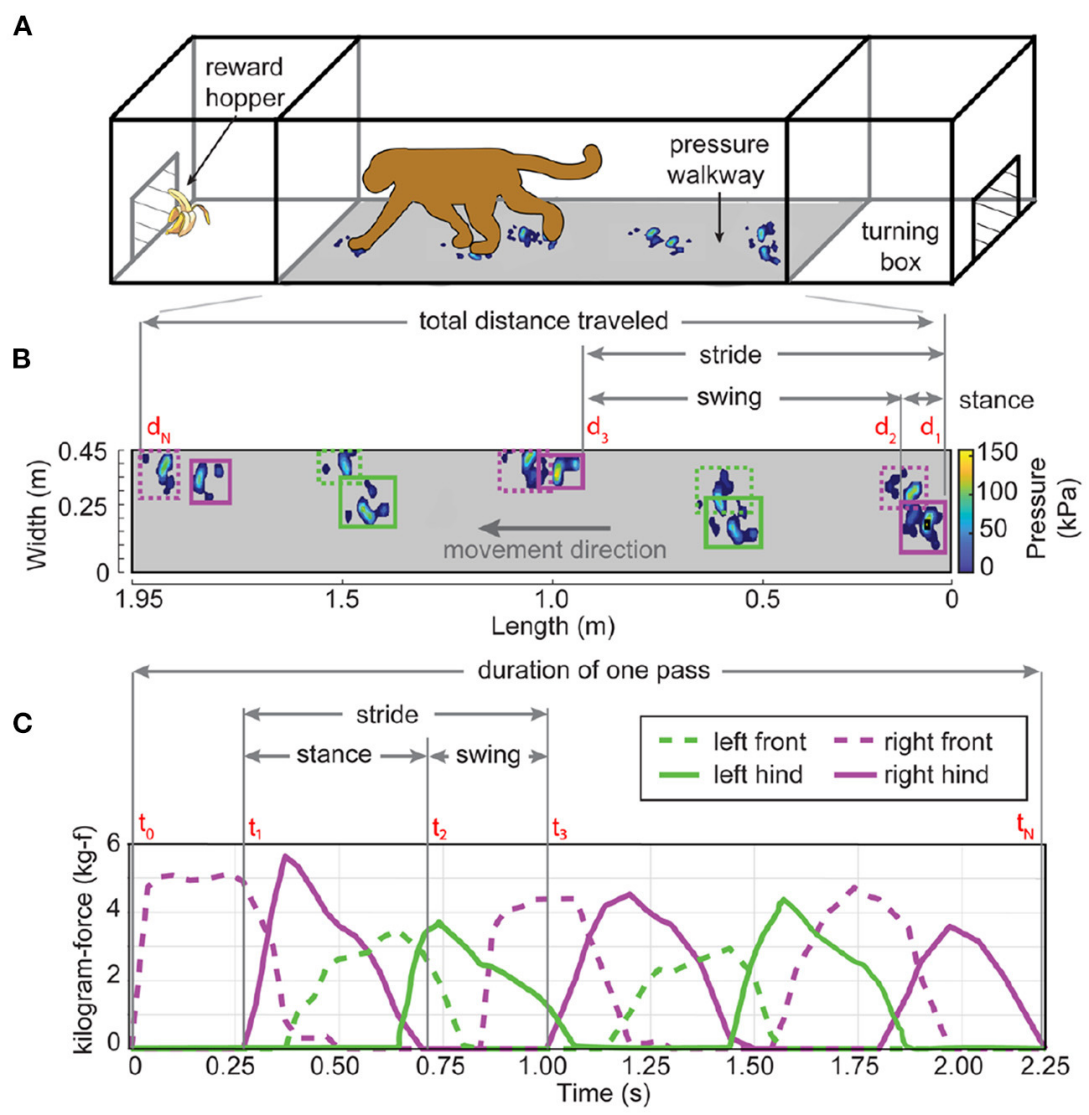
Gait testing apparatus and spatiotemporal analysis of gait parameters. **(A)** The GTA consists of a tunnel capped at each side by end enclosures with sliding doors (not shown). A pressure mat (gray shaded area) recorded location, time, and magnitude of contact pressure (pixelated blue hand and foot prints). Food or juice reward was delivered through hoppers in each end enclosure. **(B)** Overhead perspective of pixelated calibrated pressure data (color bar, kPa) recorded for a representative pass/trial from right-to-left. Post-processed ‘strike-boxes' placed around each hand or foot print identified contact points and pressure magnitudes by limb (front/hind limbs shown as dashed/solid lines, respectively, and left/right sides shown in green/purple, respectively). Interlimb distance is the length between leading edges of consecutive ipsilateral limb “strike boxes”. **(C)** Time series of the aggregate pressure data converted into kilogram-force (kg-f) captured within each ‘strike-box' defined temporal gait parameters and stance force profiles for each limb. The representation of the first stance, swing, and stride progression shown from right-to-left **(B)** is the same instance shown from left-to-right in the time series. Time and distance variables (red) are illustrated for each gait parameter, see Table 2.

Gait dysfunction following MPTP treatment was evaluated with a novel Gait Testing Apparatus (GTA). The GTA was fabricated from T-slot aluminum framing and polycarbonate paneling (80/20, Inc.) (Figure 2A). The apparatus consisted of a 2.43 m long tunnel (0.73 × 0.73 m cross-section) capped by two end enclosures (0.76 × 0.76 m cross-section) each equipped with a hopper to provide a food or juice reward. The tunnel contained a 1.95 × 0.45 m pressure walkway mat (HR Walkway 4 VersaTek system, Tekscan, Inc.) with 33,408 evenly distributed pressure sensing cells using resistive sensor technology (4 sensels/cm^2^) (Figure 2B). The pressure walkway mat was calibrated to the subject's weight prior to each recording.

**Table 2:** Definitions of spatial and temporal gait parameters.

**Gait Variable**	**Definition**
Gait Speed	The speed of one pass. The distance traveled between the heel edge of the first strike box to the toe edge of the last strike box divided by the time of the first instance of non-zero pressure data of the first strike box to the time of the last instance of non-zero pressure data in the last strike box. $\frac{d_{N}}{t_{N}}\;meters\;per\;second\;\left(m/s\right)$
Cadence (steps/min)	Step rate per pass. The number of stances within a single trail divided by trail duration and converted into steps/min. $\frac{\#Stances\;*60\;\left(seconds/minute\right)}{t_{N}}\;steps\;per\;minute\;\left(steps/min\right)$
Stride Length	The distance between two consecutive contacts of one limb (Left front heel to left front heel). The distance between the edge of the strike box closest to the heel and the edge of the next strike box closest to the heel. (*d*_3_−*d*_1_) *meters* (*m*)
Stride Time	The time between two consecutive contacts of one limb. The summation of swing time and stance time (*t*_3_−*t*_1_) *seconds* (*s*)
Swing Length	The distance the limb travels “in the air” during a stride. The distance between strike-boxes. (*d*_3_−*d*_2_) *meters* (*m*)
Swing Time	The time the limb travels while not in contact with the ground. The time between two consecutive stances when pressure was not detected. (*t*_3_−*t*_2_) *seconds* (*s*)
Stance Length	The length of a footprint. The length of the strike box. (*d*_2_−*d*_1_) *meters* (*m*)
Stance Time	The time the limb is on the ground. The time between the first and last instance of non-zero pressure mat data within a ‘strike box'. (*t*_2_−*t*_1_) *seconds* (*s*)

### Data collection

The subjects were acclimated to the GTA and trained to walk (pass) from one turning enclosure to the other for a food or juice reward. A minimum of five experimental sessions (each conducted on different days) with at least 15 successful passes were collected in the naïve state and as many passes as possible in the subsequent parkinsonian states. A successful pass was defined as the primate walking on all limbs from one enclosure to the other without stopping, turning, exhibiting excessive hesitation, or other non-continuous ambulating behaviors within the walkway. Data collection began when the subject contacted any pressure walkway sensors and stopped when the subject left the walkway. Pressure data from the walkway was digitized at 30 frames/s. Concurrent video recordings captured the profile view of the walkway.

### Data processing

“Strike-boxes” (Figure 2B, rectangle circumscribing the perimeter of each limb print) were manually placed within Tekscan software (Tekscan Walkway 7.66, Tekscan Inc.) and delineated the recorded pressure data (kPa) for each limb. This allowed for extraction of limb-specific spatiotemporal pressure data, which were exported to MATLAB (MathWorks). Custom scripts were used to calculate stance-swing phases, cadence, and gait speed. For each limb, individual stance and swing length (m) were defined by the distance within and between ‘strike-boxes', respectively (Figure 2B). Stride length (m) was the sum of the distance between consecutive stance and swing phases. Time series data of the aggregate pressure profiles (kPa) converted to kilogram-force (kgf), within and between ‘strike-boxes', defined the temporal components of gait (Figure 2C). Specifically, stance time (s) was defined as the time between the first and last instance of non-zero pressure mat data within a “strike-box”. Swing time duration was the time (s) between consecutive stances when pressure was not detected. Stride time was the sum of consecutive stance and swing times for each limb. Interlimb length was the distance between the placement of the digits of the front limb and the placement of the digits of the ipsilateral hind limb. Partial hand or footprints (< ~¾ size) near the end enclosures during the first and last stances in a pass/trial were excluded from further analysis. Additionally, the presence of a momentary pressure anywhere on the mat limited to two consecutive data frames or less (i.e., < ~67 ms) were considered noise or incomplete stances and excluded from further analysis.

Cadence, or step rate (steps/min), was defined as the number of stances (i.e., “strike-boxes” for all limbs) within a single pass/trial divided by the trial duration and then converted into steps/min. Gait speed was defined as the distance traveled (i.e., heel-to-toe distance between the first and last “strike-box” in a trial, regardless of limb) divided by the duration of the trial. A representative example of how these various measures of gait were obtained during a single right-to-left pass is shown in Figure 2B, with a total of 10 step force profiles (i.e., 10 stance “strike-boxes”) over a distance traveled of ~1.90 m (Figure 2B) and a trial duration of 2.25 s (Figure 2C). The overall cadence and gait speed for the representative pass were 267 limb steps/min (10 stances/2.25 sec × 60 sec/min) and 0.84 m/s (1.90 m/2.25 sec), respectively.

### Statistics

Within-subject behaviors were compared using a mixed model with parkinsonian “state” as fixed and ‘session nested within state' as random effects. Baseline gait parameters in the naïve state were compared across subjects using a fixed effects model with “subject” as fixed and “sessions nested within subjects” as random effects. In both models, significant main effects with more than two levels were further analyzed using a Tukey *post-hoc* test. A Levene's test for homogeneity of variance performed on swing time and length data assessed the change in variability across states. Statistical analyses were conducted in JMP (v15.2.0, SAS Institute) using standard least squares regression. Bar plots showed the least-square means (means calculated from the model) and 95% confidence intervals (CI) of the least-square means (range of 95% of the data). Bar plot 95% CIs should only be compared within animal subplots and not across animal subplots. For brevity, these values will be referred to as “means” and “95% confidence intervals of the mean” throughout the paper. Effect sizes were calculated from the least-square means and standard error from the mixed and fixed effects models using Cohen's d for unequal sample sizes and standard error. An effect size value of < 0.35 was considered a “small” effect size, 0.35–0.65 was considered “medium”, and over 0.65 was considered “large”.

## Results

### Dataset and observed behaviors

All subjects were brought from a naïve state to a moderate parkinsonian state with repeated injections of MPTP. Two of the subjects (B and A) were evaluated in a mild parkinsonian state, and two subjects were also assessed in a severely parkinsonian state (B and N) (Figure 1, Table S1). Compared to the naïve state, parkinsonian subjects showed less motivation to perform the task, less curiosity to the environment around them, and slower turns or changes in behavior. The observed change in motivation and ability to complete the task with increasing parkinsonian severity was reflected in the average number of passes per session from 21.7 (naïve 823 passes/38 sessions; mild 673 passes/31 sessions) to 10.9 (moderate 261 passes/24 sessions) to 6.2 (severe 62 passes/10 sessions) with increasing parkinsonian severity (Table S1, bottom row).

### Coordinated gait parameter changes with MPTP treatment

**Figure 3:**
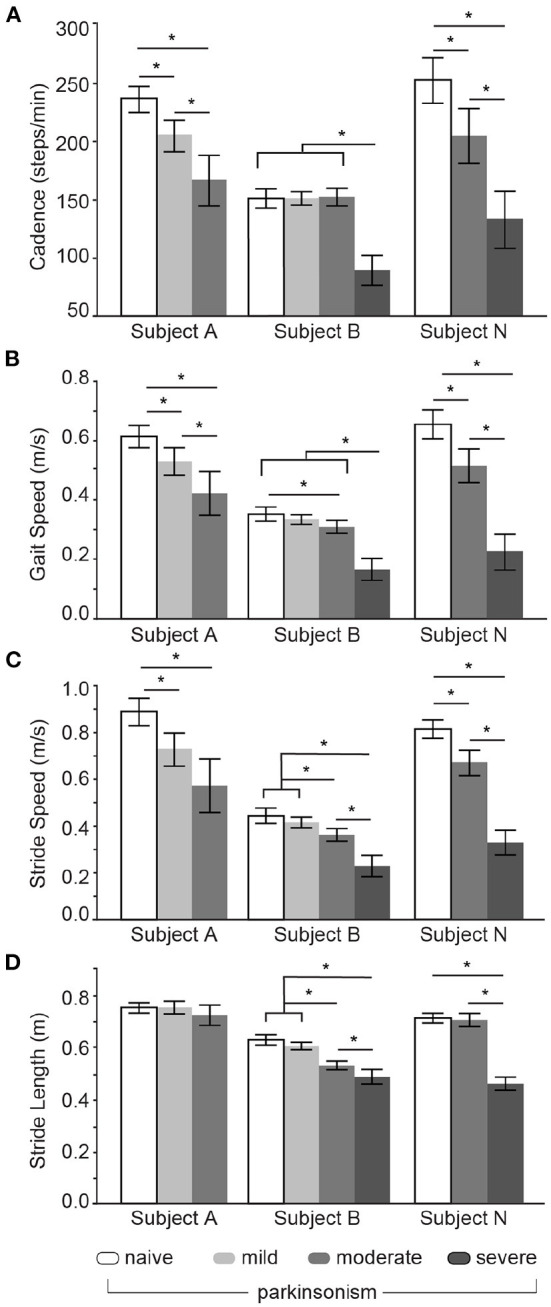
Whole-body gait parameter changes with MPTP treatment. Means and 95% CI for cadence **(A)**, gait speed **(B)**, stride speed **(C)**, and stride length **(D)** for subjects A, B, and N across naïve and parkinsonian (mild, moderate, severe) states. *denotes significance at p < 0.05 (statistical results detailed in Tables S1–S4).

Similar to humans, each subject had unique gait metrics in the baseline (MPTP naïve state; Figure 3). Subject B baseline (naïve) behavior, in particular, was considerably slower as evidenced by lower cadence, gait speed, and stride speed when compared to the other subjects in the naïve state (Figures 3A–C, white bars). The slower cadence and gait speed for subject B could not be attributed to subject body size (10, 12, 11 kgs) or age (24, 23, 16 yrs) differences amongst subjects (A, B, and N, respectively). Baseline stride length differed across subjects with longest-to-shortest stride lengths for Subject A, N, and then B (Figure 3D, white bars), but the effect size was small (Table S2). Similarity in subject size, age, and stride length support the behavioral observation that subjects A and N had a higher motivation to complete the trials compared to subject B.

Gait metrics in the naïve state steadily decreased across the parkinsonian states. Subjects A and N had significant and progressive decrements in cadence and gait speed across all parkinsonian states, whereas subject B showed either small or no changes until the moderate or severe state for cadence and gait speed (Figures 3A,B, Table S2). Stride speed and stride length for each limb were averaged within each subject and within the naïve and parkinsonian states (Figures 3C,D, respectively). All subjects exhibited slower strides across the mild, moderate, and/or severe states (Figure 3C, Table S2), aligning with an overall slowing of gait with overall increasing parkinsonian severity. In terms of stride length, there was no change in subjects A or N between the naïve and moderate states and a relatively small, but statistically significant decrease in subject B (Figure 3D, Table S2). Statistically significant and robust decreases in stride length were observed in both subjects (B, N) that reached the severe parkinsonian state.

### Stride-by-stride spatiotemporal limb dynamics

**Figure 4:**
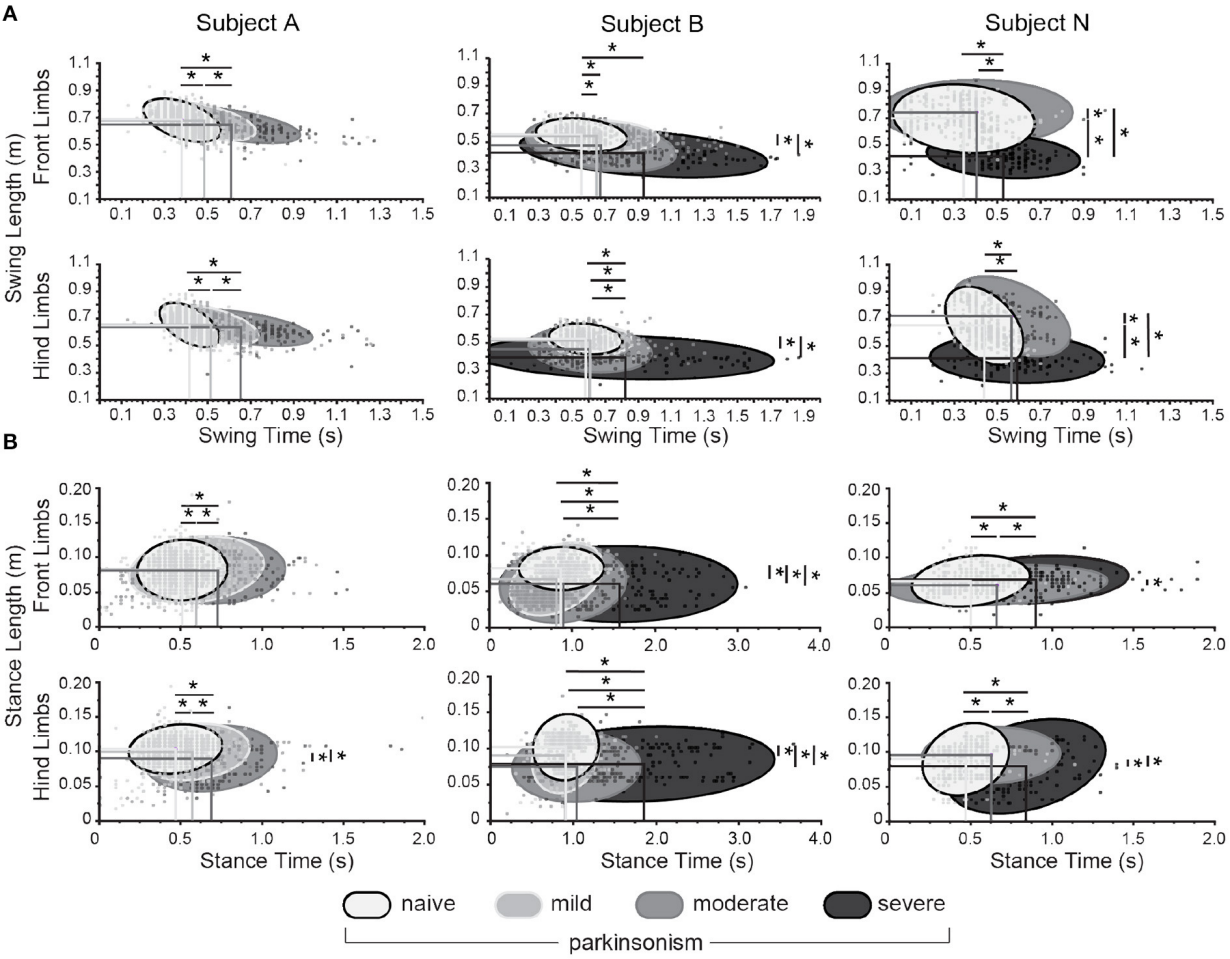
Changes in swing and stance phases between naïve and parkinsonian states. **(A)** Comparison of swing length (y axis) and swing time (x axis) of the front (top row) and hind (bottom row) limbs. Shaded ellipses represent 95% confidence intervals about the respective data set. Lines intersect at the mean swing time and swing length for the given state. **(B)** A similar comparison between stance time and stance length of the front (top row) and hind (bottom row) limbs. *denotes a significant difference (p < 0.05) of the variables between states.

To further investigate gait parameter changes with MPTP treatment, the swing and stance phases of individual strides were analyzed and compared between front and hind limb groupings. A total of 13,776 strides (Table S1) were analyzed across the three subjects in the naïve and parkinsonian states. Mean swing time (horizontal axis in Figure 4A) consistently and significantly increased for both front and hind limb groupings across parkinsonian states (see details on statistical tests and results in Table S3). In addition, the effect sizes of swing time were larger (medium or large) in the naïve to moderate/severe states than in the naïve to mild state (small) (Table S3).

Subject A showed no change in swing length (vertical axis in Figure 4A), and Subject B showed a decrease in swing length in the moderate and severe parkinsonian state with a predominantly medium effect size (Table S3). Subject N showed a decrease across all parkinsonian states with small and medium effect sizes in the moderate state and large effect sizes in the severe state (Table S3). There was also an increase in swing length and swing time variability as the subjects became more parkinsonian, as shown in Figures 4A,B by the elongation of the 95% CI ellipse in both the x- and y-direction. The increase in variance of the swing time and length was confirmed using the HOV Levene's test on aggregate limb data per subject across parkinsonian states (Table S4).

Mean stance time, shown on the horizontal axis in Figure 4B significantly increased for both front and hind limb groupings across parkinsonian states for subjects A and N. Most of the effect sizes were small in the naïve to mild and moderate states, and large in the naïve to severe state (Table S3). Mean stance length (vertical axis in Figure 4B), decreased in all parkinsonian states for subject B for the front and hind limbs, in the hind limbs in the moderate state for subject A, and in the moderate and severe State for subject N (Table S3). With the exception of subject B in the naïve to moderate state, these effect sizes were all small (Table S3).

## Discussion

This study used a novel testing apparatus to quantitatively measure volitional, naturalistic gait patterns in a preclinical MPTP non-human primate model of Parkinson's disease. The apparatus, containing a pressure walkway system comparable to those used in human studies, enabled characterization of changes in gait patterns across multiple stages of parkinsonism to identify when and to what extent spatial and temporal features of gait dysfunction occur relative to other cardinal motor signs of PD. Here, we show that NHP subjects exhibited progressive bradykinetic gait driven by a slowing of movement speed, stride speed, and increased time spent in the swing and stance phases of a stride cycle. Robust changes in stride length, on the other hand, were observed primarily in the severe parkinsonian state. Additionally, there was an increase in the variability of gait parameters with increasing parkinsonian severity.

### The gait testing apparatus for assessing gait dysfunction

This preclinical study identified high resolution changes in the spatial and temporal dynamics of gait during volitional ambulation in naïve and MPTP-treated NHPs. Prior preclinical studies investigating unconstrained walking have been limited to step-by-step spatial components of gait (18, 47), temporal measurements of gross coordinated gait behaviors (22, 23, 48), and video analysis for kinetic estimation of limb dynamics (22, 23, 49, 50). Murine studies have utilized methods such as optical imaging and ventral plane videography to not only evaluate the static and dynamic components of gait but to assess gait variables similar to those found in the human PD literature, such as base of support length, three point support (similar to double support time in bipeds) (20), and deficits in footprint intensity contralateral to the lesioned side to evaluate gait asymmetry (21). There is one study in marmoset NHPs using an automated method to measure footfalls and quantify base of support and interlimb coordination variables (31). Similar to this study, the GTA can be used in larger NHPs to quantify complex gait dynamics with precise spatial and temporal resolution. Analyzing pressure dynamics across limbs of one severe parkinsonian subject, a difference between footprint patterns, and thus disordered gait, could be ascertained in this study. Additionally, though not addressed in this current study, this approach has the potential to measure shifting of pressure dynamics within and across limbs when assessing balance and postural instability, anticipatory postural adjustments, festination (i.e., smaller but quicker steps), turning, gait initiation, and freezing of gait.

### Comparison of MPTP gait dysfunction to human PD

The most prominent sign of gait dysfunction in PD is reduced stride length (51, 52). This impaired ability of humans with PD to modulate the amplitude of their stride is thought to be the primary contributing factor for the reduction in gait speed (52–55). As observed in prior studies with MPTP treatment in NHPs, severe stage animals, like subjects B and N, had a decreased stride length (22, 23) with slower movement speed. Here, we show that while stride length can decrease slightly in the mild and moderate parkinsonian states, the largest effect on stride length occurs in the severe parkinsonian state in the MPTP-treated rhesus macaque model.

The temporal characteristics of PD gait typically includes an increase in the time it takes to complete a stride (56), with more time spent with the feet in contact with the ground (56, 57) and less time with the feet mid-air (56, 58). Overall, our subjects showed an increase in stride time and stance time; however, the time spent in the swing phase also increased. Given the decrease in gait speed, stride speed, and cadence, this is suggestive of bradykinetic gait in the NHP as opposed to one characterized by festination that can occur in some human PD cases (54).

The increase in variability of the swing phase in the parkinsonian animals is consistent with increases in variability in gait parameters found in humans with PD (12, 56, 59). While gait variability is a measure of disease severity, it is also a metric used in determining the effectiveness of therapies (60).

### Progression of gait dysfunction in the MPTP animal model

The data presented are the result of a within-subjects experimental design that is consistent with prior studies done in this field (61–63). It is well known that the MPTP NHP model captures many of the electrophysiological and phenotypic changes that occur in human PD (27, 40–43, 64), but few studies detail the parkinsonian gait deficits. NHPs in the severe state exhibit step/stride length decreases, freezing of gait, postural deficits, and difficulty turning (22–24); however, there has been little known of stride cycle changes and the appearance of gait dysfunction with increasing parkinsonian severity with NHPs.

The subjects exhibited more prominent bradykinetic gait with increasing parkinsonian severity. This matched the visual observations of subjects in the mild parkinsonian state where the gait deficits were not easily discerned as compared to the moderate or severe parkinsonian state. The GTA enabled parsing out the subtle gait changes that were more defined in later stages. For instance, subject B showed a small change in swing time of the front limbs that became more pronounced in the severe stage. This state could provide insights in the early signs of parkinsonism, as unlike with human PD studies, these gait changes can be combined with neurophysiological recordings to evaluate the pathogenic mechanisms underlying the emerging deficits and perhaps shed light into potential therapy developments for early PD. The more pronounced gait changes in the moderate and severe states are ideal for studies involving testing of novel therapeutics. In addition, the severe state provides a rich dataset for neurophysiological studies that aim to explore asymmetric gait or freezing of gait.

### Limitations and future refinements to the GTA approach

One study limitation was investigating parkinsonian gait dysfunction during ambulation in a small set of subjects. However, the relatively consistent results in the temporal gait parameters across subjects suggest that similar gait patterns will likely occur with larger cohorts of subjects. Additional subjects along the parkinsionian spectrum could further characterize subtle changes in spatial gait metrics. While the analysis focused on data collected during continuous ambulation, the GTA system could also be used to capture other gait behaviors such as freezing of gait (24), festination, difficulty turning, and obstacle maneuvering leveraging both the pressure walkway and assessment of joint kinematics through the clear polycarbonate paneling. The approach also facilitates wireless transmission of neural recordings concurrent with pressure walkway and video capture recordings, which will enable future studies to investigate the neurophysiological basis of the temporal and spatial changes in gait with increasing parkinsonian severities.

## Conclusion

This study developed a novel, quantitative approach to investigate gait dysfunction in freely-moving non-human primates (rhesus macaques) across a spectrum of parkinsonian severities. Subjects demonstrated progressive bradykinetic gait, whereas stride length changes occurred most robustly upon reaching the severe parkinsonian state. Knowing how gait metrics change with increasing parkinsonian severity will enable a broad range of translational studies to better elucidate the circuit-based pathophysiology of parkinsonian gait dysfunction (65–67) and evaluate novel pharmacological and deep brain stimulation treatments for parkinsonian gait dysfunction.

## Data availability statement

The original contributions presented in the study are included in the article/Supplementary material, further inquiries can be directed to the corresponding author.

## Ethics statement

Animal care complied with the National Institutes of Health Guide for the Care and Use of Laboratory Animals and all behavioral protocols were approved by the University of Minnesota Institutional Animal Care and Use Committee.

## Author contributions

Conceptualization: MJ, AD, JV, DB, and CH. Data curation and formal analysis and visualization: AD, DB, and CH. Funding acquisition: MJ, JV, and LJ. Investigation: AD, SN, SF, JK, LW, MB, EL, CS, DR, DS, SB, YY, AV, TH, JW, LJ, and MJ. Methodology and writing – original draft: AD, DB, CH, and MJ. Project administration and resources: MJ, JV, LJ, and JW. Software: AD, DB, KB, and ZL. Supervision: CH, MJ, JV, LJ, and JW. Writing – review and editing: AD, DB, CH, SN, SF, JK, LW, MB, EL, CS, ZL, KB, DR, DS, SB, YY, AV, TH, JW, LJ, and MJ. All authors contributed to the article and approved the submitted version.

## References

[1] Jankovic J. Parkinson's disease: clinical features diagnosis. J Neurol Neurosurg Psychiatry. (2008) 79:368–76. 10.1136/jnnp.2007.131045 18344392

[2] Lew M. Overview of Parkinson's disease. Pharmacotherapy. (2007) 27:155S−60S. 10.1592/phco.27.12part2.155S 18041935

[3] Forsaa EB Larsen JP Wentzel-Larsen T Herlofson K Alves G. Predictors and course of health-related quality of life in Parkinson's disease. Mov Disord. (2008) 23:1420–7. 10.1002/mds.22121 18512757

[4] Gomez-Esteban J Zarranz J Lezcano E Tijero B Luna A Velasco F. Influence of motor symptoms upon the quality of life of patients with Parkinson's disease. Eur Neurol. (2007) 57:161–5. 10.1159/000098468 17213723

[5] Muslimovic D Post B Speelman JD Schmand B de Haan RJ. Determinants of disability and quality of life in mild to moderate Parkinson disease. Neurology. (2008) 70:2241–7. 10.1212/01.wnl.0000313835.33830.80 18519873

[6] Uebelacker LA Epstein-Lubow G Lewis T Broughton MK Friedman JH A. survey of Parkinson's disease patients: most bothersome symptoms and coping preferences. J Parkinsons Dis. (2014) 4:717–23. 10.3233/JPD-140446 25271239

[7] Bohnen NI Albin RL Müller LTM Chou K. Advances in therapeutic options for gait and balance in Parkinson's disease. US Neurol. (2011) 7:100–8. 10.17925/USN.2011.07.02.100 24348751 PMC3859869

[8] Collomb-Clerc A Welter M-L. Effects of deep brain stimulation on balance and gait in patients with Parkinson's disease: a systematic neurophysiological review. Neurophysiol Clin. (2015) 45:371–88. 10.1016/j.neucli.2015.07.001 26319759

[9] Rascol O Payoux P Ory F Ferreira JJ Brefel-Courbon C Montastruc J-L. Limitations of current Parkinson's disease therapy. Ann Neurol. (2003) 53:S3–12. 10.1002/ana.10513 12666094

[10] Smulders K Dale ML Carlson-Kuhta P Nutt JG Horak FB. Pharmacological treatment in Parkinson's disease: effects on gait. Parkinsonism Relat Disord. (2016) 31:3–13. 10.1016/j.parkreldis.2016.07.006 27461783 PMC5048566

[11] Grabli D Karachi C Welter ML Lau B Hirsch EC Vidailhet M. Normal and pathological gait: what we learn from Parkinson's disease. J Neurol Neurosurg Psychiatry. (2012) 83:979–85. 10.1136/jnnp-2012-302263 22752693 PMC3852420

[12] Hausdorff JM Cudkowicz ME Firtion R Wei JY Goldberger AL. Gait variability and basal ganglia disorders: stride-to-stride variations of gait cycle timing in Parkinson's disease and Huntington's disease. Mov Disord. (1998) 13:428–37. 10.1002/mds.870130310 9613733

[13] Peterson DS Horak FB. Neural control of walking in people with parkinsonism. Physiology. (2016) 31:95–107. 10.1152/physiol.00034.2015 26889015 PMC4888974

[14] Vitek JL Johnson LA. Understanding Parkinson's disease deep brain stimulation: role of monkey models. Proc Natl Acad Sci U S A. (2019) 116:26259–65. 10.1073/pnas.1902300116 31871164 PMC6936402

[15] Amano S Roemmich RT Skinner JW Hass CJ. Ambulation Parkinson disease. Phys Med Rehabil Clin N Am. (2013) 24:371–92. 10.1016/j.pmr.2012.11.003 23598269

[16] Debû B De Oliveira Godeiro C Lino JC Moro E. Managing gait, balance, and posture in Parkinson's disease. Curr Neurol Neurosci Rep. (2018) 18:23. 10.1007/s11910-018-0828-4 29623455

[17] Amende I Kale A McCue S Glazier S Morgan JP Hampton TG. Gait dynamics in mouse models of Parkinson's disease and Huntington's disease. J NeuroEngineering Rehabil. (2005) 2:20. 10.1186/1743-0003-2-20 16042805 PMC1201165

[18] Fernagut PO Diguet E Labattu B Tison F. A simple method to measure stride length as an index of nigrostriatal dysfunction in mice. J Neurosci Methods. (2002) 113:123–30. 10.1016/S0165-0270(01)00485-X 11772434

[19] Goldberg RS Hampton T McCue S Kale A Meshul CK. Profiling changes in gait dynamics resulting from progressive 1-methyl-4-phenyl-1,2,3,6-tetrahydropyridine-induced nigrostriatal lesioning. J Neurosci Res. (2011) 89:1698–706. 10.1002/jnr.22699 21748776

[20] Wang XH Lu G Hu X Tsang KS Kwong WH Wu FX. Quantitative assessment of gait and neurochemical correlation in a classical murine model of Parkinson's disease. BMC Neurosci. (2012) 13:142. 10.1186/1471-2202-13-142 23151254 PMC3507899

[21] Vandeputte C Taymans J-M Casteels C Coun F Ni Y Van Laere K. Automated quantitative gait analysis in animal models of movement disorders. BMC Neurosci. (2010) 11:92. 10.1186/1471-2202-11-92 20691122 PMC2924851

[22] Grabli D Karachi C Folgoas E Monfort M Tande D Clark S. Gait disorders in parkinsonian monkeys with pedunculopontine nucleus lesions: a tale of two systems. J Neurosci. (2013) 33:11986–93. 10.1523/JNEUROSCI.1568-13.2013 23864685 PMC6794061

[23] Karachi C Grabli D Bernard FA Tandé D Wattiez N Belaid H. Cholinergic mesencephalic neurons are involved in gait and postural disorders in Parkinson disease J. Clin Invest. (2010) 120:2745–54. 10.1172/JCI42642 20628197 PMC2912198

[24] Revuelta GJ Uthayathas S Wahlquist AE Factor SA Papa SM. Non-human primate FOG develops with advanced parkinsonism induced by MPTP Treatment. Exp Neurol. (2012) 237:464–9. 10.1016/j.expneurol.2012.07.021 22967858 PMC3582410

[25] Chassain C Eschalier A Durif F. Assessment of motor behavior using a video system a clinical rating scale in parkinsonian monkeys lesioned by MPTP. J Neurosci Methods. (2001) 111:9–16. 10.1016/S0165-0270(01)00425-3 11574115

[26] Luquin MR Montoro RJ Guillén J Saldise L Insausti R Del Río J. Recovery of chronic parkinsonian monkeys by autotransplants of carotid body cell aggregates into putamen. Neuron. (1999) 22:743–50. 10.1016/S0896-6273(00)80733-3 10230794

[27] Ovadia A Zhang Z Gash DM. Increased susceptibility to MPTP toxicity in middle-aged rhesus monkeys. Neurobiol Aging. (1995) 16:931–7. 10.1016/0197-4580(95)02012-8 8622784

[28] Goetz L Piallat B Bhattacharjee M Mathieu H David O Chabardès S. On the role of the pedunculopontine nucleus and mesencephalic reticular formation in locomotion in nonhuman primates. J Neurosci. (2016) 36:4917–29. 10.1523/JNEUROSCI.2514-15.2016 27147647 PMC6601854

[29] Larson SG. Unique Aspects of Quadrupedal Locomotion in Nonhuman Primates, In:, editors, E Strasser, JG Fleagle, AL Rosenberger AL, *Primate Locomotion: Recent Advances*. (Boston, MA: Springer), pp 157–173. (1998).

[30] Wells JP Turnquist JE. Ontogeny of locomotion in rhesus macaques (*Macaca mulatta*): IPostural I, and locomotor behavior and habitat use in a free-ranging colony. Am J Phys Anthropol. (2001) 115:80–94. 10.1002/ajpa.1059 11309753

[31] Pickett KA Schultz-Darken N Bradfield AF Malicki K Pape B Ausderau KK. Spatiotemporal quantification of gait in common marmosets. J Neurosci Methods. (2020) 330:108517. 10.1016/j.jneumeth.2019.108517 31730871 PMC7012682

[32] Hass CJ Malczak P Nocera J Stegemöller EL Wagle Shukla A Malaty I. Quantitative normative gait data in a large cohort of ambulatory persons with Parkinson's disease. PLoS ONE. (2012) 7:e42337. 10.1371/journal.pone.0042337 22879945 PMC3411737

[33] Kim CM Eng JJ. Symmetry in vertical ground reaction force is accompanied by symmetry in temporal but not distance variables of gait in persons with stroke. Gait Posture. (2003) 18:23–8. 10.1016/S0966-6362(02)00122-4 12855297

[34] Merory JR Wittwer JE Rowe CC Webster KE. Quantitative gait analysis in patients with dementia with Lewy bodies and Alzheimer's disease. Gait Posture. (2007) 26:414–9. 10.1016/j.gaitpost.2006.10.006 17161601

[35] Vallabhajosula S Humphrey SK Cook AJ Freund JE. Concurrent validity of the zeno walkway for measuring spatiotemporal gait parameters in older adults. J Geriatr Phys Ther. (2019) 42:E42–50. 10.1519/JPT.0000000000000168 29286982

[36] Youdas JW Hollman JH Aalbers MJ Ahrenholz HN Aten RA Cremers JJ. Agreement between the GAITRite walkway system and a stopwatch-footfall count method for measurement of temporal and spatial gait parameters. Arch Phys Med Rehabil. (2006) 87:1648–52. 10.1016/j.apmr.2006.09.012 17141647

[37] Halliday SE Winter DA Frank JS Patla AE Prince F. The initiation of gait in young, elderly, and Parkinson's disease subjects. Gait Posture. (1998) 8:8–14. 10.1016/S0966-6362(98)00020-4 10200394

[38] Mancini M Weiss A Herman T Hausdorff JM. Turn around freezing: community-living turning behavior in people with Parkinson's disease. Front Neurol. (2018) 9:18. 10.3389/fneur.2018.00018 29434567 PMC5790768

[39] Kappos EA Sieber PK Engels PE Mariolo AV D'Arpa S Schaefer DJ. Validity and reliability of the CatWalk system as a static and dynamic gait analysis tool for the assessment of functional nerve recovery in small animal models. Brain Behav. (2017) 7:e00723. 10.1002/brb3.723 28729931 PMC5516599

[40] Johnson MD Vitek JL McIntyre CC. Pallidal stimulation that improves parkinsonian motor symptoms also modulates neuronal firing patterns in primary motor cortex in the MPTP-treated monkey. Exp Neurol. (2009) 219:359–62. 10.1016/j.expneurol.2009.04.022 19409895 PMC2730829

[41] Langston JW. The MPTP story. J Parkinsons Dis. (2017) 7:S11–9. 10.3233/JPD-179006 28282815 PMC5345642

[42] Masilamoni GJ Smith Y. Chronic MPTP administration regimen in monkeys: a model of dopaminergic non-dopaminergic cell loss in Parkinson's disease. J Neural Transm. (2018) 125:337–63. 10.1007/s00702-017-1774-z 28861737 PMC5826821

[43] William Langston J Forno LS Rebert CS Irwin I. Selective nigral toxicity after systemic administration of 1-methyl-4-phenyl-1,2,5,6-tetrahydropyrine (MPTP) in the squirrel monkey. Brain Res. (1984) 292:390–4. 10.1016/0006-8993(84)90777-7 6607092

[44] Connolly AT Muralidharan A Hendrix C Johnson L Gupta R Stanslaski S. Local field potential recordings in a non-human primate model of Parkinsons disease using the activa PC + S neurostimulator. J Neural Eng. (2015) 12:066012. 10.1088/1741-2560/12/6/066012 26469737 PMC5130227

[45] Hendrix CM Campbell BA Tittle BJ Johnson LA Baker KB Johnson MD. Predictive encoding of motor behavior in the supplementary motor area is disrupted in parkinsonism. J Neurophysiol. (2018) 120:1247–55. 10.1152/jn.00306.2018 29873615 PMC6171054

[46] Vitek JL Zhang J Hashimoto T Russo GS Baker KB. External pallidal stimulation improves parkinsonian motor signs and modulates neuronal activity throughout the basal ganglia thalamic network. Exp Neurol. (2012) 233:581–6. 10.1016/j.expneurol.2011.09.031 22001773 PMC3536483

[47] Tsai YF Tsai HW Tai MY Lu KS. Age-related changes in locomotor behavior induced by MPTP in rats. Neurosci Lett. (1991) 129:153–5. 10.1016/0304-3940(91)90743-D 1922966

[48] Campos-Romo A Ojeda-Flores R Moreno-Briseño P Fernandez-Ruiz J. Quantitative evaluation of MPTP-treated nonhuman parkinsonian primates in the HALLWAY task. J Neurosci Methods. (2009) 177:361–8. 10.1016/j.jneumeth.2008.10.026 19022292

[49] Broom L Ellison BA Worley A Wagenaar L Sörberg E Ashton C. A translational approach to capture gait signatures of neurological disorders in mice and humans. Sci Rep. (2017) 7:3225. 10.1038/s41598-017-03336-1 28607434 PMC5468293

[50] Geldenhuys WJ Guseman TL Pienaar IS Dluzen DE Young JW A. novel biomechanical analysis of gait changes in the MPTP mouse model of Parkinson's disease. PeerJ. (2015) 3:e1175. 10.7717/peerj.1175 26339553 PMC4558067

[51] Galna B Lord S Burn DJ Rochester L. Progression of gait dysfunction in incident Parkinson's disease: impact of medication and phenotype. Mov Disord. (2015) 30:359–67. 10.1002/mds.26110 25546558

[52] Morris ME Iansek R Matyas TA Summers JJ. Ability to modulate walking cadence remains intact in Parkinson's disease. J Neurol Neurosurg Psychiatry. (1994) 57:1532–4. 10.1136/jnnp.57.12.1532 7798986 PMC1073238

[53] Knutsson E. An analysis of Parkinsonian gait. Brain. (1972) 95:475–86. 10.1093/brain/95.3.475 4655275

[54] Morris ME Iansek R Matyas TA Summers JJ. The pathogenesis of gait hypokinesia in Parkinson's disease. Brain. (1994) 117:1169–81. 10.1093/brain/117.5.1169 7953597

[55] Morris ME Iansek R Matyas TA Summers JJ. Stride length regulation in Parkinson's disease: Normalization strategies and underlying mechanisms. Brain. (1996) 119:551–68. 10.1093/brain/119.2.551 8800948

[56] Blin O Ferrandez AM Serratrice G. Quantitative analysis of gait in Parkinson patients increased variability of stride length. J Neurol Sci. (1990) 98:91–7. 10.1016/0022-510X(90)90184-O 2230833

[57] Ferrandez A-M Blin O A. comparison between the effect of intentional modulations and the action of l-Dopa on gait in Parkinson's disease. Behav Brain Res. (1991) 45:177–83. 10.1016/S0166-4328(05)80083-X 1789925

[58] Hausdorff JM Lowenthal J Herman T Gruendlinger L Peretz C Giladi N. Rhythmic auditory stimulation modulates gait variability in Parkinson's disease. Eur J Neurosci. (2007) 26:2369–75. 10.1111/j.1460-9568.2007.05810.x 17953624

[59] Hausdorff JM Schaafsma JD Balash Y Bartels AL Gurevich T Giladi N. Impaired regulation of stride variability in Parkinson's disease subjects with freezing of gait. Exp Brain Res. (2003) 149:187–94. 10.1007/s00221-002-1354-8 12610686

[60] Su ZH Patel S Gavine B Buchanan T Bogdanovic M Sarangmat N. Deep brain stimulation and levodopa affect gait variability in Parkinson disease differently. Neuromodul Technol Neural Interf. (2022) 11:S1094715922006353. 10.1016/j.neurom.2022.04.035 35562261

[61] Campbell BA Cho H Faulhammer RM Hogue O Tsai JP Hussain MS Machado AG Baker KB. Stability and effect of parkinsonian state on deep brain stimulation cortical evoked potentials. Neuromodul Technol Neural Interf. (2021) 25:ner.13508. 10.1111/ner.13508 34309115 PMC10246651

[62] Choudhury GR Daadi MM. Charting the onset of Parkinson-like motor and non-motor symptoms in nonhuman primate model of Parkinson's disease. PLoS ONE. (2018) 13:e0202770. 10.1371/journal.pone.0202770 30138454 PMC6107255

[63] Wichmann T Bergman H Starr PA DeLong MR Watts RL Subramanian T. Comparison of MPTP-induced changes in spontaneous neuronal discharge in the internal pallidal segment and in the substantia nigra pars reticulata in primates. Exp Brain Res. (1999) 125:397–409. 10.1007/s002210050696 10323285

[64] Porras G Li Q Bezard E. Modeling Parkinson's disease in primates: the MPTP model. Cold Spring Harb Perspect Med. (2012) 2:a009308. 10.1101/cshperspect.a009308 22393538 PMC3282499

[65] Galvan A Devergnas A Wichmann T. Alterations in neuronal activity in basal ganglia-thalamocortical circuits in the parkinsonian state. Front Neuroanat. (2015) 9:5. 10.3389/fnana.2015.00005 25698937 PMC4318426

[66] Karachi C Francois C. Role of the pedunculopontine nucleus in controlling gait and sleep in normal and parkinsonian monkeys. J Neural Transm. (2018) 125:471–83. 10.1007/s00702-017-1678-y 28084536

[67] Goetz L Piallat B Bhattacharjee M Mathieu H David O Chabardès S. Spike discharge characteristic of the caudal mesencephalic reticular formation and pedunculopontine nucleus in MPTP-induced primate model of Parkinson disease. Neurobiol Dis. (2019) 128:40–8. 10.1016/j.nbd.2018.08.002 30086388

