# Supplementary material for: Spatiotemporal scaling changes in gait in a progressive model of Parkinson's disease
Source: Front Neurol. 2022 Dec 13;13:1041934. doi: 10.3389/fneur.2022.1041934 (PMC9792983; doi:10.3389/fneur.2022.1041934)
Supplement: Supplementary file 1 [file Table_1.DOCX]

# **Supplementary Tables**

**Table S1.** Summary of pressure walkway data collection. Number of recording sessions (experimental days) and total number of observations (passes and strides per state) are listed for each subject. Cells with ‘.’ indicate unavailable data.

|  | Naïve | | | Mild Parkinsonism | | | Moderate Parkinsonism | | | Severe Parkinsonism | | |
| --- | --- | --- | --- | --- | --- | --- | --- | --- | --- | --- | --- | --- |
| Subject | **Session #** | **# of passes** | **# of strides** | **Session #** | **# of passes** | **# of strides** | **Session #** | **# of passes** | **# of strides** | **Session #** | **# of passes** | **# of strides** |
| A | 19 | 565 | 3652 | 13 | 308 | 1988 | 5 | 120 | 838 | . | . | . |
| B | 9 | 122 | 1007 | 18 | 365 | 3332 | 14 | 100 | 946 | 5 | 23 | 312 |
| N | 10 | 136 | 885 | . | . | . | 5 | 41 | 231 | 5 | 39 | 585 |
| *Subtotal* | ***38*** | ***823*** | ***5544*** | ***31*** | ***673*** | ***5320*** | ***24*** | ***261*** | ***2015*** | ***10*** | ***62*** | ***897*** |

## **Table S2:** Cadence, gait speed, stride speed, and stride length statistics for all subjects across parkinsonian states.

|  |  |  | **Parkinsonian States** | | | | **Statistic** | |
| --- | --- | --- | --- | --- | --- | --- | --- | --- |
|  |  | **Subject** | **Naïve**  **LS Mean, Std. Error, N** | **Mild**  **LS Mean, Std. Error, N** | **Moderate**  **LS Mean, Std. Error, N** | **Severe**  **LS Mean, Std. Error, N** | **State Effect, Post Hoc** | **Effect size (Cohen’s D)** |
| **A.**  **Cadence** | | B | 150, 4, 122 | 150, 3, 365 | 152,4,100 | 89,6,23 | F (3,41) =28.32,  p < 0.0001*  Post HOC: naïve & mild & moderate > severe | naïve > severe: 1.4318 |
|  |  | A | 236, 5, 565 | 205, 7, 305 | 167, 11, 120 |  | F (2,34) = 19.03,  p < 0.0001*  Post HOC: naïve > mild > moderate | naïve > mild: 0.2515, naïve > moderate: 0.5468 |
|  |  | N | 252,9,136 |  | 205,11,41 | 133,12,39 | F (2,16) = 32.45  p < 0.0001*  Post HOC: naïve > moderate > severe | naïve > moderate: 0.4768, naïve > severe: 1.191 |
| **B.**  **Gait Speed** | | B | 0.35,0.01,122 | 0.33, 0.01,365 | 0.31,0.01,100 | 0.17,0.02,23 | F (3,41) = 26.79  p < 0.0001*  Post HOC: naïve > moderate > severe | naïve > moderate: 0.3589  naïve > severe: 1.5148 |
|  |  | A | 0.62, 0.02, 565 | 0.53,0.02, 305 | 0.42,0.04,120 |  | F (2,34) = 12.76,  p < 0.0001*  Post HOC: naïve > mild > moderate | naïve > mild: 0.2044, naïve > moderate: 0.4486 |
|  |  | N | 0.66,0.02,136 |  | 0.52,0.03,41 | 0.22,0.03,39 | F (2,16) = 69.84,  p <0.0001*  Post HOC: naïve > moderate > severe | naïve > moderate: 0.5566, naïve > severe: 1.752 |
| **C.**  **Stride Speed** | | B | 0.44,0.02,1007 | 0.42,0.01,3332 | 0.36,0.01,946 | 0.23,0.02,312 | F (3,40) =23.61  p <0.0001*  Post HOC: naïve & mild > moderate > severe | naïve > moderate: 0.1742  naïve > severe: 0.4365 |
|  |  | A | 0.88,0.03,3652 | 0.72,0.03,1988 | 0.57,0.06,838 |  | F (2,34) = 14.54,  p<0.0001*  Post HOC: naïve> mild & moderate | naïve > mild: 0.094, naïve > moderate: 0.1817 |
|  |  | N | 0.81,0.02,885 |  | 0.67,0.03,231 | 0.32,0.02,585 | F (2,14) = 126.82  p <0.0001*  Post HOC: naïve > moderate > severe | naïve > moderate: 0.2763, naïve > severe: 0.8567 |
| **D.**  **Stride Length** | | B | 0.63,0.01,1007 | 0.60,0.01,3332 | 0.53,0.01,946 | 0.49,0.01,312 | F (3,40) =39.15,  p <0.0001*  Post HOC: naïve & mild > moderate > severe | naïve > moderate: 0.3418,  naïve > severe: 0.4692 |
|  |  | A | 0.75,0.01,3652 | 0.75,0.01,1988 | 0.72,0.02,838 |  | F (2,34) =0.95,  p = 0.40 |  |
|  |  | N | 0.71,0.01,885 |  | 0.70,0.01,231 | 0.46,0.01,585 | F (2,13) = 168.97  p <0.0001*  Post HOC: naïve & moderate > severe | naïve > severe: 0.9412 |
| **E.**  **Stride length, naïve state, across subjects** | | B | 0.63,0.01,1007 |  |  |  | F(2,35) = 30.84, p <0.0001*  Post HOC: Subject B < Subject N < Subject A | Subject A >  Subject N: 0.0791  Subject A > Subject B: 0.2409,  Subject N > Subject B: 0.2057, |
|  |  | A | 0.75,0.01,3652 |  |  |  |  |  |
|  |  | N | 0.71,0.01,986 |  |  |  |  |  |

*denotes significant differences across states at p < 0.05. If more than one state was present, a post-hoc Tukey test was used and is indicated as ‘Post HOC’ in the table with significantly different states displayed on a scale.

## **Table S3:** Swing phase and stance phase statistics for all subjects across parkinsonian states.

|  |  |  |  | **Parkinsonian States** | | | | **Statistic** | |
| --- | --- | --- | --- | --- | --- | --- | --- | --- | --- |
|  |  | **Subject** | **Limb** | **Naïve**  **LS Mean, Std. Error, N** | **Mild**  **LS Mean, Std. Error, N** | **Moderate**  **LS Mean, Std. Error, N** | **Severe**  **LS Mean, Std. Error, N** | **State Effect** | **Effect size (Cohen’s D)** |
| **A.**  **Swing Length** | | A | F | 0.67,0.01,1797 | 0.67,0.01,991 | 0.64,0.02,413 |  | F (2,34) = 0.94,  p = 0.40 |  |
|  |  |  | H | 0.65,0.01,1855 | 0.65,0.01,997 | 0.63,0.02,425 |  | F (2,34) = 0.44,  p = 0.65 |  |
|  |  | B | F | 0.55,0.01,494 | 0.54,0.01,1653 | 0.48,0.01,472 | 0.43,0.02,154 | F (3,38) = 19.18,  p <0.0001*  Post HOC:  naïve & mild > moderate > severe | Naive > moderate: 0.2813  naïve > severe: 0.4975 |
|  |  |  | H | 0.52,0.01,513 | 0.51,0.01,1679 | 0.45,0.01,474 | 0.41,0.01,156 | F (3,39) = 28.05,  p <0.0001*  Post HOC:  naïve & mild > moderate > severe | Naive > moderate: 0.3556  naïve > severe: 0.5762 |
|  |  | N | F | 0.68,0.01,427 |  | 0.74,0.01,112 | 0.40,0.01,297 | F (2,15) = 195.97  p <0.0001*  Post HOC:  moderate > naïve > severe | moderate > naïve: 0.3007, naïve > severe: 1.270 |
|  |  |  | H | 0.63,0.01,458 |  | 0.72,0.02,119 | 0.39,0.01,288 | F (2,14) = 135.10  p <0.0001*  Post HOC:  moderate > naïve > severe | moderate > naïve: 0.3723, naïve > severe: 0.981 |
| **B.**  **Swing Time** | | A | F | 0.38,0.01,1797 | 0.48, 0.01,991 | 0.60,0.02,413 |  | F (2, 34) = 50.57  p <0.0001*,  Post HOC: moderate > mild > naïve | naïve > mild: 0.2248, naïve > moderate: 0.4986 |
|  |  |  | H | 0.42,0.01,1855 | 0.51,0.01,997 | 0.65,0.02,425 |  | F (2,34) = 60.89,  p < 0.0001*  Post HOC: moderate > mild > naïve | naïve > mild: 0.228, naïve > moderate: 0.5486 |
|  |  | B | F | 0.56,0.02,494 | 0.65,0.01,1653 | 0.67,0.01,472 | 0.90, 0.03,154 | F (3,35) = 44.30,  p <0.0001*  Post HOC:  severe > mild & moderate > naïve | naïve > mild: 0.2155, naïve > moderate: 0.3231  naïve > severe: 0.9651 |
|  |  |  | H | 0.58,0.01,513 | 0.61,0.01,1679 | 0.61,0.01,474 | 0.81,0.02,156 | F (3,41) = 28.97  p < 0.0001* | naïve > severe: 0.7289 |
|  |  | N | F | 0.37,0.02,427 |  | 0.41,0.02,112 | 0.55,0.02,297 | F (2,15) =24.48  P <0.0001*  Post HOC: severe > moderate & naïve | naïve > severe: 0.5214 |
|  |  |  | H | 0.43,0.02,458 |  | 0.57,0.02,119 | 0.62,0.02,288 | F (2,18) = 30.41  P <0.0001*  Post HOC: severe & moderate > naïve | naïve > moderate: 0.4163, naïve > severe: 0.5605 |
| **C.**  **Stance Length** | | A | F | 0.08,0.01,1797 | 0.09,0.01,991 | 0.08,0.01,413 |  | F(2,34) = 0.17, p = 0.84 |  |
|  |  |  | H | 0.10,0.01,1855 | 0.10,0.01,997 | 0.09,0.01,425 |  | F(2,34) = 12.87, p < 0.001*, Post HOC: naïve & mild > moderate | naïve > moderate: 0.2604 |
|  |  | B | F | 0.08,0.00,494 | 0.07,0.00,1653 | 0.06,0.00,472 | 0.06,0.00,154 | F(3,47) = 16.01, p < 0.0001*, Post HOC: naïve > mild & moderate & severe | naïve > mild: 0.2232, naïve > moderate: 0.4175  naïve > severe: 0.4062 |
|  |  |  | H | 0.10, 0.00,513 | 0.09, 0.00,1679 | 0.08,0.00,474 | 0.08,0.00,156 | F(3,50) = 30.03, p < 0.0001*, Post HOC: naïve > mild > moderate & severe | naïve > mild: 0.1839, naïve > moderate: 0.5644  naïve > severe: 0.4620 |
|  |  | N | F | 0.07,0.01,45 |  | 0.06,0.01,112 | 0.07,0.01,297 | F(2,17) = 5.11, p < 0.05*, Post HOC: naïve > moderate | naïve > moderate: 0.2749 |
|  |  |  | H | 0.09,0.01,50 |  | 0.09,0.01,119 | 0.08,0.01,288 | F(2,17) = 10.45, p < 0.01*,Post HOC: moderate & naïve > severe | naïve > severe: 0.1371 |
| **D.**  **Stance Time** | | A | F | 0.51,0.02,1797 | 0.61,0.02,991 | 0.72,0.03,413 |  | F(2,34) = 16.23, p < 0.0001*, Post HOC: naïve < mild < moderate | naïve > mild: 0.1307, naïve > moderate, 0.2806 |
|  |  |  | H | 0.47,0.02,1855 | 0.58,0.02,997 | 0.68,0.04,425 |  | F(2,34) = 16.19, p < 0.001*, Post HOC: naïve < mild < moderate | naïve> mild: 0.1388, naïve>moderate: 0.2696 |
|  |  | B | F | 0.88,0.04,494 | 0.82,0.03,1653 | 0.88,0.03,472 | 1.53,0.06,154 | F(3,32) = 45.93, p < 0.0001*  severe > naïve & mild & moderate | naïve > severe: 0.7916 |
|  |  |  | H | 0.91,0.05,513 | 0.91,0.04,1679 | 1.02,0.04,474 | 1.77,0.07,156 | F(3,37) =38.50, p < 0.0001*, Post Hoc: severe > moderate & naïve & mild | naïve > severe: 0.7453 |
|  |  | N | F | 0.50,0.02,427 |  | 0.66,0.02,112 | 0.90,0.02,297 | F(2,19) = 113.82, p <0.001*, Post HOC: naïve < moderate < severe | naïve > moderate: 0.476, naïve>severe: 1.1373 |
|  |  |  | H | 0.48,0.02,458 |  | 0.62,0.03,119 | 0.86,0.03,288 | F(2,18) = 66.38, p < 0.0001*, Post HOC: naïve < moderate < severe | naïve > moderate: 0.3414, naïve > severe: 0.8723 |
| **E.**  **Stride Time** | | A | F | 0.89,0.03,1797 | 1.09,0.03,991 | 1.32,0.05,313 |  | F(2,34) = 32.31, p < 0.0001*, Post HOC: naïve < mild < moderate | naïve > mild:0.182, naïve > moderate:0.397 |
|  |  |  | H | 0.89,0.03 | 1.09,0.03,997 | 1.33,0.05,425 |  | F(2,34) = 32.62, p < 0.0001*, Post HOC: naïve < mild < moderate | naïve > mild: 0.182, naïve > moderate: 0.392 |
|  |  | B | F | 1.43,0.06,494 | 1.47,0.04,1653 | 1.54,0.05,472 | 2.41,0.08,154 | F(3,34) =42.41, p < 0.0001*, Post HOC: severe > moderate & mild & naïve | naïve > severe: 0.8387 |
|  |  |  | H | 1.50,0.06,513 | 1.52,0.04,1679 | 1.63,0.05,474 | 2.57,0.09,156 | F(3,36) = 41.60, p < 0.0001*, Post HOC: severe > moderate & mild & naïve | naïve > moderate: 0.7889 |
|  |  | N | F | 0.87,0.03,427 |  | 1.06,0.04,112 | 1.44,0.03,297 | F(2,17) = 94.79, p < 0.0001*, Post HOC: naïve < moderate < severe | naïve > moderate: 0.378, naïve > severe: 1.047 |
|  |  |  | H | 0.92,0.03,458 |  | 1.20,0.05,119 | 1.49,0.04,288 | F(2,17) = 53.75, p < 0.0001*, Post HOC: naïve < moderate < severe | naïve > moderate: 0.398, naïve > severe: 0.784 |

## **Table S4:** Tests of variability of swing phase.

|  |  |  | **Parkinsonian State** | | | | **Statistic** |
| --- | --- | --- | --- | --- | --- | --- | --- |
|  |  | **Subject** | **Naïve**  **Stdev, N** | **Mild**  **Stdev, N** | **Moderate**  **Stdev, N** | **Severe**  **Stdev, N** | **Levene Test** |
| **A.**  **Swing Length** | | B | 0.05,1007 | 0.05,3332 | 0.07,946 | 0.07, 312 | naïve/mild: F(1,4337)=0.41, p=0.52  mild/moderate: F(1,4276)=301.95, p<0.0001* moderate/severe: F(1,1256) = 6.07, p = 0.0139* |
|  |  | A | 0.07,3652 | 0.06,1988 | 0.06,838 |  | naïve/mild: F(1,5638) = 38.76, p<0.0001*  mild/moderate: F(1,2824) = 10.35, p =0.0013* |
|  |  | N | 0.11,885 |  | 0.11,231 | 0.07,585 | naïve/moderate: F(1,1114) = 0.13, p = 0.72  moderate/severe: F(1,814) = 96.44, p<0.0001* |
| **B.**  **Swing Time** | | B | 0.10,1007 | 0.13,3332 | 0.17,946 | 0.35,312 | naïve/mild: F(1,4337) = 55.77, p<0.0001*  mild/moderate: F(1,4276) = 103.26, p<0.0001* |
|  |  | A | 0.07,3652 | 0.10,1988 | 0.14,838 |  | naïve/mild: F(1,5638) = 144.30, p<0.0001*  mild/moderate: F(1,2824)=95.26, p<0.0001* |
|  |  | N | 0.12,885 |  | 0.17,231 | 0.16,585 | naïve/moderate: F(1,1114) = 39.18, p<0.0001*  moderate/severe: F(1,814) = 1.13, p = 0.29 |
